# Enduring Clinical Value of Copaxone® (Glatiramer Acetate) in Multiple Sclerosis after 20 Years of Use

**DOI:** 10.1155/2019/7151685

**Published:** 2019-01-15

**Authors:** Daniel R. Wynn

**Affiliations:** Director, Clinical Research; Consultants in Neurology, Multiple Sclerosis Center, Northbrook, IL, USA

## Abstract

Multiple sclerosis (MS) is a chronic progressive neurodegenerative demyelinating disease affecting the central nervous system. Glatiramer acetate (GA; Copaxone®) was the first disease-modifying treatment (DMT) for MS successfully tested in humans (1977) and was approved by the US Food and Drug Administration in December 1996. Since then, there have been numerous developments in the MS field: advances in neuroimaging allowing more rapid and accurate diagnosis; the availability of a range of DMTs including immunosuppressant monoclonal antibodies and oral agents; a more holistic approach to treatment by multidisciplinary teams; and an improved awareness of the need to consider a patient's preferences and patient-reported outcomes such as quality of life. The use of GA has endured throughout these advances. The purpose of this article is to provide an overview of the important developments in the MS field during the 20 years since GA was approved and to review clinical data for GA in MS, with the aim of understanding the continued and widespread use of GA. Both drug-related (efficacy versus side-effect profile and monitoring requirements) and patient factors (preferences regarding mode of administration and possible pregnancy) will be explored.

## 1. Introduction

Multiple sclerosis (MS) is a chronic progressive neurodegenerative demyelinating disease affecting the central nervous system (CNS). Symptoms of MS include fatigue, visual impairment, spasticity, ataxia, tremor, bladder/bowel dysfunction, sexual dysfunction, pain, and cognitive impairment. These symptoms have a negative impact on patients' quality of life (QOL) as a consequence of reduced independence, ability to work, and participation in social/leisure activities [1]. MS is a highly individual disease with a different course in each patient.

Interferon beta-1b (Betaseron®) was the first Food and Drug Administration (FDA)-approved disease-modifying treatment (DMT) for MS in 1993, followed by Avonex® (interferon beta-1a) in May 1996, and Copaxone® (glatiramer acetate [GA]) in December 1996. Generic versions of GA are now available; however, throughout this article, “GA” will be used to refer to Copaxone®. A discussion of the development of generic versions is beyond the scope of this article and the reader is referred elsewhere [2–4].

**Figure 1:**
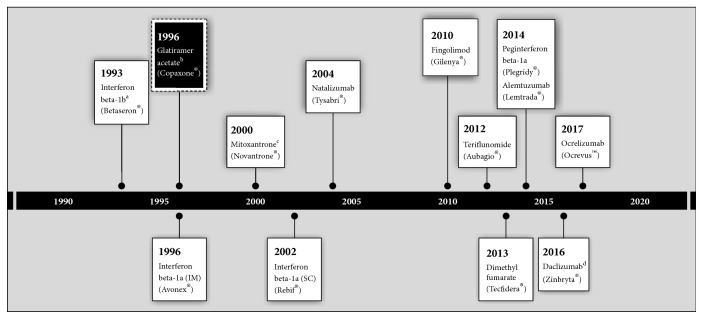
Timeline of approval by the FDA of disease-modifying therapies for multiple sclerosis. FDA: Food and Drug Administration; IM: intramuscular; SC: subcutaneous. ^a^Interferon beta-1b was also approved in 2009 as Extavia® (which is the Novartis-branded version of the Bayer product Betaseron®). ^b^Various generic versions of glatiramer acetate are in development. Glatopa™ [10] was approved in 2015 by the FDA. Other generic versions were approved in the EU in 2016 and by the FDA in 2017 [11, 12]. ^c^Bioequivalent generic mitoxantrone was approved in 2006. ^d^Subsequently withdrawn (March 2018).

GA was the first subsequently approved DMT successfully tested in humans [5, 6] and it has been studied for over 40 years. GA has a complex mechanism of action that is not fully understood; however, both neuroprotective and immunomodulatory effects are thought to be involved (reviewed by Comi et al. [7]). Since the introduction of the interferon betas and GA, a wide variety of DMTs have been approved, including the immunosuppressant monoclonal antibodies natalizumab, alemtuzumab, daclizumab (subsequently withdrawn, March 2018), and ocrelizumab (Figure 1). Despite this, use of GA has endured. Indeed, GA has been the most commonly prescribed DMT for relapsing MS in the USA since 2008 (40 mg and 20 mg combined prescriptions; based on prescriptions for the market definition of DMT for relapsing forms of MS in the US [8]). Interestingly, in a study of 102 predominantly US neurologists, although efficacy was considered the most important attribute of a DMT by neurologists, GA was the most commonly prescribed [9]. It should also be noted that the development of multiple generic versions of GA provides further support for the continued clinical value of GA in MS.

The purpose of this article is to explore the reasons behind the enduring use of GA through narrative review of the important developments in the MS field during the 20 years since GA was approved and a summary of the key data for GA in MS.

## 2. Developments in the MS Field Over the Last Two Decades

There have been significant developments in the field of MS during the two decades since the approval of GA, which are summarized below. Before discussing this, however, it is relevant to look further back in history. The study of currently available DMTs for MS began 40 years ago, with the first clinical investigation of GA [5], which led to the development of modern-day MS trial design. This first GA study [5] initiated a cascade of research, which resulted in both improved study design and treatment options for patients with MS. In parallel during this period, the evolution of magnetic resonance imaging (MRI) technology has allowed the dynamic inflammatory processes in MS to be viewed* in vivo*. Over this period, assessments of MS developed considerably, from focusing on number and severity of relapses, to also considering disability worsening, through to use of MRI endpoints such as number of new brain lesions, whole brain volume, and brain atrophy, and finally to determining segmental brain volume changes (reviewed in detail elsewhere [13]). There has also been a growing appreciation that changes in CNS gray matter occur in MS [14], which has permitted a more in-depth understanding and prediction of the course of MS in an individual.

In the 1970s, the average time to diagnosis was 7 years [15]; a definite diagnosis by an MS specialist is now usually provided within 6 months of referral [16]. This has been facilitated by refinement in MRI technology and the consequent changes to the MS diagnostic criteria (2010 and 2017 Revisions to the McDonald criteria [17, 18]). The 2017 revisions to the McDonald criteria allow for a diagnosis (and therefore treatment) of MS in patients with a typical clinically isolated syndrome and clinical or MRI demonstration of dissemination in space and presence of cerebrospinal fluid-specific oligoclonal bands [18]. However, given the low symptom burden in these patients, consideration of the tolerability of a DMT is particularly important during decision-making.

An understanding of the critical role played by B cells (reviewed by von Büdingen et al. [19]) is another important advance in the MS field. This, together with initial reports of the efficacy of the CD20-targeting B-cell-depleting agent rituximab in MS [20, 21], has paved the way for the development of anti-B-cell therapies, such as ocrelizumab, which is FDA approved for both relapsing MS and primary progressive MS, for which it was the first FDA-approved therapy (March 2017). Additional agents, e.g., ofatumumab and ublituximab, are currently in Phase 3 clinical trials. To date, however, there is no universally accepted treatment algorithm for MS [22].

Together, the improvements in diagnosis and availability of effective treatment options have allowed earlier treatment of patients. The consensus of opinion supports initiation of DMTs early in the course of MS [22, 23] with the goal of preventing accumulation of irreversible neurological damage and worsening to secondary progressive MS. Attention has now turned to consideration of personalized therapy, with a goal of establishing the most effective and safe treatment outcomes.

Two treatment approaches are currently used for initial treatment of MS: lower- versus higher-efficacy DMTs from the time of disease diagnosis. Administration of lower-efficacy therapies at disease diagnosis is more common and is suitable for patients who present with MS with favorable prognostic factors. It involves initial use of therapies such as GA and interferon betas, followed by newer agents (dimethyl fumarate, fingolimod, teriflunomide, natalizumab, alemtuzumab, ocrelizumab, etc.) if the patient's response to first-line treatment is suboptimal. In contrast, administration of high-efficacy therapies from the time of MS diagnosis is used for patients with aggressive MS where the risk of early permanent disability from active disease may outweigh the risk of drug-related safety issues (detailed later). This approach involves early use of immunosuppressive therapy to achieve disease control, after which patients may switch to another agent if there is either lack of efficacy, or tolerability or safety concerns. The best approach for early treatment is the topic of considerable debate and is beyond the scope of this article; the subject has been extensively reviewed elsewhere [24–26]. Key points of the debate are the differences in risk:benefit for the disease versus adverse drug effects.

Such has been the progress in treatment of MS that it is now possible to contemplate the target of “no evident disease activity” (NEDA), i.e., the absence of clinical disease activity (relapse, disease progression, and radiographic lesions determined by MRI). Although this is currently a topic of much debate, a discussion on the applicability of NEDA is beyond the scope of this review. In the author's opinion, the issues with NEDA at present [27] limit its usefulness in clinical practice. Furthermore, repeatedly switching DMTs in an attempt to achieve NEDA, in the absence of a cure for MS, may result in both unknown safety consequences and unclear benefits in preventing long-term disability.

Implementation of the management of MS by multidisciplinary teams has resulted in substantial improvements in the experience of patients with MS [28, 29]. Care is now delivered by teams of clinicians including a wide range of specialists. A further positive development in MS management is multidisciplinary pharmacological management of the disabling symptoms of MS (reviewed by Toosy et al. [30]).

Although not unique to MS, consideration of patients' views on disease management has contributed to improved patient care. Shared decision-making between the patient and physician is now considered best practice [31] and is thought to improve treatment satisfaction and adherence [32]. Associated with the importance of patients' views is the recognition of the importance of patient-reported outcomes in MS [1]. In the absence of a curative therapy, and given the different safety profiles of DMTs, shared decision-making and patient-reported outcomes are particularly important for the treatment of MS and affect treatment adherence (discussed later). Obtaining patient-reported outcome data is now standard in MS clinical trials.

Further areas of interest in the field of MS that are beyond the scope of this review but are attracting a considerable degree of attention include the potential involvement of the gut microbiome [33]; ongoing efforts to identify pharmacogenetic markers for treatment response [34]; and the possibility of cell-based therapies for MS (particularly neuroprotection and/or repair of MS-related damage to the CNS) [35].

Although progress has been made across the MS field during the past 20 years, a range of treatment issues remain to be addressed. No targeted DMT is approved for secondary progressive MS, although the Phase 3 EXPAND study reported that siponimod reduced the risk of disability progression in patients with secondary progressive MS [36]. Furthermore, as a consequence of the availability of numerous DMTs, treatment decisions are now more complex. However, reliable biological markers for predicting future disease course and response to treatment are not yet available.

## 3. The Clinical Value of Glatiramer Acetate in the Treatment of MS

GA (also known as copolymer-1) was designed as a synthetic analog of myelin basic protein, a presumptive autoantigen associated with MS. GA is a standardized mixture of polypeptides randomly polymerized from four l-amino acids found in myelin basic protein: l-glutamic acid, l-lysine, l-alanine, and l-tyrosine, in a defined molar residue ratio of 0.14:0.34:0.43:0.09 [7]. The average molecular mass of GA is 5–9 kDa [37]. GA was initially approved as a 20 mg subcutaneous (SC) once-daily formulation; it is now also available as a 40 mg three-times weekly formulation. Possible reasons for the long-standing use of GA in the treatment of MS will be explored through review of the clinical data for GA and factors (relating to both the drug and patient) affecting the choice of DMTs for MS.

### 3.1. Clinical Data for Glatiramer Acetate

#### 3.1.1. Pivotal Clinical Studies and Long-Term Data

GA was initially investigated in a case series [5], followed by two small pilot studies [6, 38]. Two pivotal placebo-controlled studies subsequently demonstrated the efficacy of GA in patients with relapsing MS (US Glatiramer Acetate Trial [39] and the European/Canadian MRI study [40]; see Table 1).

The 2-year US Glatiramer Acetate Trial was the US registration study and reported a 29% reduction in the annualized relapse rate (ARR) for GA treatment compared with placebo. Similarly, the 9-month European/Canadian MRI study reported a 33% reduction in the relapse rate [40]. These short-term studies did not report significant improvements in disability progression as assessed by the Expanded Disability Status Scale (EDSS). However, the US Glatiramer Acetate Trial open-label extension study reported consistently low ARRs (15 years: 0.25; 20 years: 0.2) with more than three-quarters of patients being ambulatory without mobility aids (EDSS score <6) at 15 years (82%) and 20 years (79.5%) [41, 42]. Furthermore, 65% and 53% had not progressed to secondary progressive MS at the 15- and 20-year timepoints, respectively [41, 42]. These data are for patients who continued in the study (n=100 at 15 years; n=74 at 20 years); as with all long-term extension studies, the issue of selection bias owing to drop-outs of patients with more aggressive disease should be borne in mind when considering these data. Overall, the two long-term extension studies (Table 1) demonstrated that the efficacy of GA is maintained over time [41–44]. Furthermore, there are no reports of a rebound effect or delayed reactivation following discontinuation [41, 44, 48, 49]. As detailed in Table 1, real-world data from a variety of sources (retrospective studies, US claims database analysis, and propensity score-matched analysis of the MSBase registry) support the efficacy data reported in clinical studies [46–49].

Lower-frequency dosing regimens have been investigated for GA. A large Phase 3 placebo-controlled trial (GALA study [58]) in GA-naïve patients reported similar safety and efficacy profiles for GA 40 mg three-times weekly compared with the earlier pivotal controlled trials of a 20 mg once-daily dose. This trial led to FDA approval in January 2014 of the GA three-times weekly SC formulation (40 mg). A further study (GLACIER) demonstrated the efficacy, safety, and tolerability of switching from GA 20 mg SC once daily (after >6 months' therapy) to 40 mg three-times weekly [59].

In both pivotal trials of GA 20 mg, injection-site reaction was the most common adverse event associated with GA treatment [39, 40] and safety in the extension studies was consistent with the placebo-controlled phases, with no long-term safety issues identified [41–44]. The three-times weekly formulation was noted to be associated with a 50% reduction in the annualized risk of injection-site reactions compared with the once-daily formulation (GLACIER study [59]). To date, GA is the only DMT with more than 10 years of continuously monitored safety data, with follow-up data for up to 20 years also available (US Glatiramer Acetate Trial [41, 42]; see Table 1). It is interesting to note that patients in the extension study were committed to self-administering daily SC injections of GA, thus emphasizing the long-term tolerability and patient acceptance of GA and its route of administration [41].

Importantly, results from a comprehensive database analysis [60] of the long-term safety and tolerability of GA in all patients with MS who have ever been exposed to GA (20 mg SC daily) in clinical trials are consistent with the long-term extension studies. In brief, the total exposure to GA was 10 017 patient-years and treatment duration ranged from 0 to 23.1 years (median 1.8 years). Injection-site-related events were the most common adverse events, affecting 49% of patients; erythema at the injection site was the most common effect (29%). Such local injection-site reactions are generally transient, resolving within hours to days and decreasing in frequency over time [61]. An acute and transient immediate systemic postinjection reaction including at least one of the following symptoms: flushing, chest tightness, palpitations, and dyspnea, is also common; the database analysis found an incidence of 24%, with dyspnea being the most common manifestation (12%). Other common adverse events were rash (15%), headache (14%), infection (12%), and vasodilation (11%) [60]. No unexpected adverse events were recorded [60]. The prescribing information for GA includes a warning that localized lipoatrophy and, rarely, injection-site necrosis may occur with GA treatment [37]. Lipoatrophy (loss of fat tissue resulting in depressions in the skin) at injection sites may occur up to several months after treatment initiation and persist after treatment cessation [62]. Although some studies suggest that it occurs in up to 64% of patients treated with GA [62], the database analysis found it to be reported as an adverse event in 0.3% of GA-treated patients [60]. Patients are advised to rotate injection sites daily to assist in minimizing such effects [37].

#### 3.1.2. MRI Data

The European/Canadian Glatiramer Acetate MRI study was designed to evaluate the effect of GA on MRI-monitored features of MS and included monthly MRI scans. This study reported a 29% reduction in the number of gadolinium-enhancing lesions with GA treatment and treatment effects favoring GA across other MRI endpoints [40]. Although MRI endpoints were not included in the initial placebo-controlled phase of the US Glatiramer Acetate Study [39], MRI evaluation was added during the open-label long-term follow-up [63]. GA treatment had modest but consistent effects in reducing brain atrophy metrics compared with placebo [63]. Overall, available MRI data suggest that GA treatment reduces brain axonal metabolic injury, tissue damage, atrophy, and brain volume loss [45, 64–67].

#### 3.1.3. Comparator Studies

Few head-to-head studies have compared GA 20 mg with other DMTs (Table 2). In general, similar clinical efficacy has been observed to that of interferon beta-1a and interferon beta-1b (Table 2). In short-term studies, there are conflicting data on brain volume loss with GA versus interferons [51, 53] (see Table 2). A long-term study of brain volume changes reported that patients receiving GA experienced a significantly lower reduction in brain volume over 5 years compared with those receiving low-dose interferon beta-1a or high-dose interferon beta-1b [45]. However, these data should be interpreted with caution as MRI parameters were not standardized across studies.

A systematic Cochrane review comparing GA with interferon treatments for MS reported similar efficacy at 24 months for clinical endpoints (number of patients with relapse, interferon versus GA: risk ratio [RR] 1.04, 95% confidence interval [CI] 0.87 to 1.24; and worsening [EDSS progression]: RR 1.11, 95% CI 0.91 to 1.35; both moderate-grade evidence) and some MRI endpoints (new gadolinium-enhancing T1 lesions: mean difference [MD] interferon versus GA -0.14, 95% CI -0.30 to 0.02; moderate-grade evidence); however, long-term data (>3 years) were not included [68]. In contrast, when MRI lesion load accrual was analyzed, interferon treatments were found to limit the increase in lesion burden to a greater extent than GA (total T2-weighted lesion volume MD -0.58, 95% CI -0.99 to -0.18, p=0.004; total T1-weighted lesion volume MD -0.20, 95% CI -0.33 to -0.07, p=0.003; moderate-grade evidence) [68].

A 2-year Phase 3 study designed to compare GA 20 mg and dimethyl fumarate (240 mg two- [FDA-approved dose] or three-times daily) with placebo reported that estimated treatment effects for clinical and MRI outcomes were numerically similar for the active comparators or greater for dimethyl fumarate [54]. Although of limited value, a post hoc direct comparison suggested a significantly greater treatment effect for dimethyl fumarate (twice-daily dose) versus GA for the number of new or enlarging hyperintense lesions on T2-weighted images [54].

#### 3.1.4. Switching to Glatiramer Acetate and Combining with Glatiramer Acetate

Although GA is an established first-line treatment option, its use as a second-line option, e.g., when the patient experiences lack of efficacy or suboptimal response, adverse events, or development of neutralizing antibodies to interferons, has also been explored in patients with relapsing-remitting MS. Reductions in relapse rates, as well as improvements in fatigue, have been reported for patients switched – because of suboptimal efficacy or intolerable adverse events – to GA from interferon beta-1a and -1b [69–73]. Switching to GA has also been explored as a possible option for patients discontinuing natalizumab owing to concerns about progressive multifocal leukoencephalopathy (PML). A few studies have examined this option in small numbers of patients. Although some investigators reported insufficient disease control following a switch to GA [74, 75], others concluded that there was no evidence of rebound disease and that switching to GA because of the risk of PML with natalizumab treatment was a potential option when the risk:benefit balance is taken into account [76, 77]. A potential weakness of any switching study is the “regression to the mean” phenomenon, relevant for switches owing to lack of efficacy of the previous treatment [7]. Further limitations are that treatment adherence tends to improve following a switch of treatment, and that in the case of MS, studies are usually too short for a meaningful comparison of efficacy owing to the nonlinear nature of the disease. It is important to note that results of large randomized controlled studies that investigate switching to GA are not yet available.

GA's safety profile favors the possibility of combining it with other DMTs in patients with relapsing MS; however, there is the potential for interactions between the mechanisms of action of GA and other drugs to significantly affect efficacy and safety [78]. A randomized, placebo-controlled, double-blind, 6-month Phase 2 safety study that investigated the addition of natalizumab 300 mg every 4 weeks to GA 20 mg/day reported some improvement in MRI metrics compared with GA alone [79]. There were no unexpected safety issues other than an increased persistence of natalizumab neutralizing antibodies in patients receiving the drug combination compared with observations in previous studies of natalizumab. Similarly, a randomized, placebo-controlled, double-blind, 48-week, Phase 2 study of the addition of teriflunomide 7 or 14 mg/day to GA 20 mg/day reported acceptable safety and tolerability and some improvement in MRI outcomes with the combination compared with GA alone [80].

One of the most likely combinations to be administered in clinical practice is that of GA with interferon betas. The CombiRx trial was a randomized, placebo-controlled, double-blind Phase 3 study that investigated the efficacy and safety of GA 20 mg/day plus interferon beta-1a 30 *μ*g once weekly compared with each drug alone [55]. Three-year data demonstrated no benefit in clinical outcomes for the combination compared with GA alone. Compared with interferon alone, the combination showed a significant reduction in ARR, although no differences were seen in confirmed EDSS progression. Some MRI outcomes were improved with the combination versus the monotherapies; no unexpected safety issues were noted. Similar results were reported after a further 4 years of follow-up [56].

Several other agents, including minocycline, intravenous steroids, estriol, and albuterol, have been investigated in combination with GA in small studies [81–85]. All these combinations have been reported to be well tolerated and to show potential for improved outcomes but require further investigation.

#### 3.1.5. Patient-Reported Outcome Data

A positive impact of GA on three key aspects of QOL – physical (disability, strength), psychological (fatigue, depression, and cognitive function), and social functioning (daily activities) – has been reported in patients with MS [41, 42, 71, 86–89]. Importantly, the positive effects of GA on QOL appear to be sustained, as shown by evidence from the 2-year FOCUS study and its extension [90, 91], and a 6-year analysis of data from the UK [92].

Physical disability, fatigue, depression, and incontinence together may contribute to loss of employment. In an analysis of insurance claims for patients receiving GA or interferon beta therapy, only GA was associated with a reduction in the total number of days missed from work over the previous year (reduction of 54 days [93]). In addition, GA was associated with reductions in patient-reported fatigue ratings and days missed from work [94]. It should be noted, however, that a recent Cochrane review comparing GA and interferons [68] stated that there are insufficient data for a comparison of GA and interferons with regard to patient-reported outcomes; thus, these data should be interpreted with caution. Furthermore, there are limited comparative data on patient-reported outcomes for GA versus orally or intravenously administered therapies.

#### 3.1.6. Pregnancy

Pregnancy data from small case series and small country-specific registries indicate that GA appears to be without teratogenic effect [95–100]. These limited studies are supported by an analysis of data on over 7000 pregnancies (collected over more than 20 years) exposed to GA [101, 102]. Analysis of this large database demonstrated that exposure to GA during pregnancy does not increase the risk of pregnancy loss compared with reference pregnancy-loss rates in the general population [101]. The authors of the report stated that these data provide further support for GA as the drug of choice for women of child-bearing age with MS who consider pregnancy [101]. Pregnancy outcomes from the GA database have also been compared with data from two external reference sources: EUROCAT (a European network of population-based registries for the epidemiologic surveillance of congenital anomalies) and the Metropolitan Atlanta Congenital Defects Program (a US population-based system). Importantly, pregnancies exposed to GA were not at higher risk for congenital anomalies than reference rates in the general population [102]. The authors concluded that, “GA exposure during pregnancy appears safe and without teratogenic effect” [102]. In addition, data from the GA database on pregnancy outcomes for women with MS who were exposed to GA during all three trimesters have been compared with that for the general population (EUROCAT) [103]. The authors concluded that “GA exposure during all three trimesters did not significantly increase the risk of congenital anomalies” [103]. The pregnancy contraindication was removed from the EU label in December 2016 [104]. In the US, GA is FDA Pregnancy Category B (although pregnancy categories are being phased out by the FDA, these are still cited in the prescribing information) [37].

In summary, the wealth of clinical and real-world data for GA, together with its positive effects on QOL, including fatigue and depression, support the efficacy and benefits of GA for patients with MS. The extensive long-term safety data and pregnancy rating also provide peace of mind for physicians and patients alike.

### 3.2. Twenty Years On, What Is the Current Role of Glatiramer Acetate in MS in Clinical Practice?

The decision to begin DMT for patients with MS is complex, owing to the need for long-term treatment and the range of available DMTs. Drug characteristics (efficacy versus side-effect profile and monitoring requirements) together with patient factors (comorbidities, contraindications, preferences regarding mode of administration, and possible pregnancy for women) need to be carefully considered, particularly as long-term treatment will be necessary.

#### 3.2.1. Drug Factors

A wide variety of DMTs are available for the treatment of MS and the efficacy and adverse-event profiles of each drug should be carefully explained to patients to allow informed decision-making. In comparator studies and network meta-analysis, GA has demonstrated similar or better efficacy compared with interferon betas (Table 2) [105]. No head-to-head studies have been reported against the more recently developed DMTs. A network meta-analysis of published data for US-approved MS DMTs reported that only alemtuzumab (RR 0.56, 95% CI 0.46 to 0.67), mitoxantrone (RR 0.56, 95% CI 0.32 to 0.98), and natalizumab (RR 0.67, 95% 0.55 to 0.82) demonstrated significantly greater effectiveness than GA in reducing the ARR over 24 months and only alemtuzumab (RR 0.46, 95% CI 0.33 to 0.65) was significantly more effective with regard to disability worsening over 24 months [105]. In other comparisons the 24-month ARR was lower with GA compared with teriflunomide and dimethyl fumarate, and the relative risk of disability worsening was lower for GA compared with teriflunomide and fingolimod, but these failed to reach statistical significance. It is important to note, however, that when considering efficacy data from MS trials conducted from the 1980s through to the present day, comparison between DMTs is complicated by the observation of a decrease in ARRs in MS study populations receiving placebo during this time period (analyzed by Inusah et al. [106]). It is also important to note that although maximizing efficacy is an important goal for consideration during decision-making, patient preference, safety, and insurance coverage often dictate the final treatment choice.

During discussion of the safety profiles of each drug with a patient, contraindications, comorbid conditions, and possible drug interactions should be considered on a case-by-case basis via review of drug-specific prescribing information. Regarding clinical safety, interferon betas are associated with a variety of adverse events including flu-like symptoms, liver function abnormalities, and injection-site reactions [107–110]. For GA, the most commonly reported adverse event is injection-site reaction.

A recent systematic review aimed to test the number needed to treat to benefit (NNTB) and to harm (NNTH), and the likelihood to be helped or harmed (LHH) when assessing benefits, risks, and benefit:risk ratios for DMTs [111]. These metrics are useful to physicians as the data are provided in a clinically relevant form. The authors reported that 4 patients (95% CI 3 to 10 patients) needed to be treated with GA rather than placebo to prevent one relapse over 2 years; the NNTB for GA was not improved compared with placebo for disability progression [111]. In terms of benefit:risk ratios (i.e., LHH), based on NNTH for adverse events leading to discontinuation of the study drug and NNTB based on ARR, GA had the most favorable LHH (59.0) compared with the other first-line DMTs [111].

In contrast to interferon betas and GA, newer agents do not benefit from the availability of similar long-term safety data. Furthermore, several of the newer agents are associated with serious adverse events (as detailed in product-specific prescribing information) including: PML (natalizumab, dimethyl fumarate, and fingolimod); infections (natalizumab, alemtuzumab, ocrelizumab, fingolimod, and daclizumab); liver toxicity (natalizumab, teriflunomide, dimethyl fumarate, fingolimod, and daclizumab); hypersensitivity reactions (natalizumab, daclizumab, and dimethyl fumarate); infusion reactions (alemtuzumab and ocrelizumab); cytopenia (alemtuzumab); lymphopenia (dimethyl fumarate); macular edema (fingolimod); posterior reversible encephalopathy syndrome (fingolimod); respiratory effects (fingolimod); cardiovascular effects (fingolimod and mitoxantrone); malignancies (fingolimod, alemtuzumab, ocrelizumab, and mitoxantrone); and autoimmune/immune disorders (alemtuzumab and daclizumab). Fatal adverse events remain rare with careful treatment monitoring; however, the risks for these events should be carefully explained to patients. The vital nature of postapproval pharmacovigilance was highlighted recently by the case of daclizumab (liver toxicity and encephalitis) and previously with cases of PML associated with natalizumab, fingolimod, and dimethyl fumarate.

Postmarketing surveillance for the use of GA in patients with MS has not shown a risk for opportunistic infections (including PML) [60]. GA does not appear to be associated with risks for abnormal liver function, thyroid disease, leukopenia, or the development of neutralizing antibodies [37, 60]. No deaths have been associated with GA treatment [60]. Furthermore, results from existing clinical trials do not suggest any significant interactions between GA and other therapies commonly used in patients with MS, including the concurrent use of corticosteroids for up to 28 days; however, interactions between GA and other drugs have not been fully evaluated [37]. Owing to the possible safety issues detailed above, most DMTs have monitoring requirements before, during, and after therapy. For example, use of interferon betas requires monitoring of complete blood count, and thyroid and liver function, as well as screening for depression [107]; mitoxantrone requires yearly quantitative left ventricular ejection fraction evaluation after stopping the drug [112]; use of fingolimod necessitates monitoring for infection during treatment and for 2 months after discontinuation [113]; administration of natalizumab must be accompanied by monitoring for PML [114]; and alemtuzumab requires monthly blood monitoring until 48 months after the last infusion [115]. Premedication and/or concomitant medications are not needed with GA and there are no monitoring requirements. Furthermore, safety/tolerability is not compromised if doses are missed; however, good injection practice and medication adherence should be verified.

In summary, considering the wealth of clinical data available for GA, good tolerability and the well-characterized safety profile make GA an attractive first-line option for patients with relapsing MS including clinically isolated syndrome.

#### 3.2.2. Patient Factors

Once the decision to start a DMT is made, because long-term treatment will be necessary, consideration of patient preference in terms of drug characteristics (e.g., mode of administration, efficacy, and safety), future pregnancy, and postdrug monitoring requirements is essential to ensure satisfaction with treatment and adherence to therapy. The issue of treatment cost and access needs to be taken into account; however, this is complex and beyond the scope of this review.

DMTs for MS are administered via infusion, self-injection (SC or intramuscular), or orally. Infusion-based therapies require a clinical visit and can be associated with infusion reactions (e.g., alemtuzumab [115], natalizumab [114], and ocrelizumab [116]). Self-injectable therapies require training in good injection technique and may be associated with injection-site reactions. Injectable therapies may be less suitable for patients with needle phobia. Daily oral medication is reported to be preferable to other routes of administration for patients with MS [117, 118] and is suggested to improve adherence [9]. However, in the author's opinion, an overlooked advantage of injectable therapies, such as GA, is that it can be easier for a patient to remember self-injecting medication compared with swallowing a tablet. Also, injectable therapies may be preferable in patients with intestinal conditions in which absorption of oral drugs may be compromised (e.g., irritable bowel syndrome, Crohn's disease, bariatric surgery, etc.). Such patients are not included in clinical trials of oral drugs, so the effects of oral agents in patients with these conditions are unknown. Injectable therapies also provide assurance regarding bioavailability, while some orally administered DMTs (such as dimethyl fumarate) are affected by food. Ingestion of a high-fat, high-calorie meal reduced the absorption of dimethyl fumarate (maximum plasma concentration [C_max_] was reduced by 40% and time taken to achieve C_max_ was delayed from 2.0 to 5.5 hours [119]).

In a discrete-choice experiment assessing DMTs in patients with MS (n=125), the most important attribute was reported by patients to be side effects of therapy (relative importance: 50%), followed by delay in disease progression (relative importance: 19%) and route and frequency of administration (relative importance: 14%) [118]. Similarly, a choice-based conjoint-analysis study reported that severe side effects had the biggest impact on patient preference for a DMT for MS [120]. A further study of patients with MS naïve to oral DMTs identified liver toxicity and serious side effects as the most significant drivers of DMT selection [121]. Together, these studies highlight the significance of drug safety to patients when making a therapy decision.

High frequency of daily dosing and certain side effects (e.g., hair thinning, risk in pregnancy, severe side effects, and diarrhea) were reported by patients to be the most important barriers to DMT adherence [121]. Similarly, a prospective multicenter real-life observational study (n=520) reported that the most frequent reason for discontinuing treatment was adverse events/side effects; route of administration, i.e., oral versus injectable, was not a significant predictor of persistence with first-line DMT [122]. Interestingly, in an 18-year observational cohort study, GA therapy was associated with a high rate of persistence with therapy [123]. Although not stated, GA administration in this study is assumed to have been via once-daily 20 mg injections as the study ended before approval of the 40 mg three times a week GA formulation. Similar adherence rates have been reported for GA and oral DMTs (fingolimod, teriflunomide, and dimethyl fumarate) [124]. A network meta-analysis demonstrated that the relative risk of withdrawal due to adverse events in patients receiving GA 20 mg was lower than in those receiving interferon beta-1a 30 or 44 *μ*g, interferon beta-1b 500 *μ*g, or dimethyl fumarate 240 mg twice- or three-times daily, although it was higher than in those receiving interferon beta-1b 250 *μ*g [125]. The three-times weekly GA formulation was reported to be associated with numerical improvements in patients' perceptions of treatment convenience compared with the once-daily formulation (GLACIER study [59]).

Some DMTs are accompanied by patient-support programs, which involve a multidisciplinary approach with contributions from patients, healthcare professionals, and pharmaceutical companies, e.g., Shared Solutions® for Copaxone® and Extracare Program for Extavia®. Such programs are particularly important during the first months following therapy initiation for injectable DMTs to minimize injection-site reactions and other side effects. In addition, these programs provide ongoing education and support for the duration of treatment, improving therapy adherence [126, 127]. These services are funded by pharmaceutical companies. Potential loss of such services with the substitution of generic formulations of DMTs is, in the author's opinion, a concern for both clinicians and patients.

Pregnancy information is particularly important with drug therapy for MS as most patients starting DMTs are women of child-bearing potential. It is critical to discuss family planning with women (including the possibility of unplanned pregnancy and breastfeeding), alongside methods of contraception. Indeed, failing to warn women of potential teratogenic effects of medications is a common reason for litigation in the USA. GA is differentiated from all other DMTs by its pregnancy category. Other DMTs are known to be found in semen (e.g., teriflunomide [128]) or breast milk (e.g., natalizumab [114], mitoxantrone [112], and ocrelizumab [although the prescribing information states there are no data on the presence of ocrelizumab in human milk, ocrelizumab was excreted in the milk of ocrelizumab-treated monkeys] [116]); therefore, these factors should be taken into account during treatment-decision discussions with all patients. Given the high rate of unplanned pregnancy, a further consideration is that some DMTs (natalizumab and fingolimod) have been associated with disease rebound following discontinuation [129, 130].

For all MS DMTs except GA, monitoring is required for clinical/safety reasons, as mentioned earlier; however, it is a potential inconvenience to patients and their family/caregivers. Patients need to understand the commitment and time required by the monitoring requirements for each DMT. In addition, explanation of the rationale for monitoring is critical for patient compliance. For example, although the incidence of some adverse events is low (e.g., PML), monitoring is essential due to the serious/potentially fatal outcomes. The costs of frequent monitoring add significant expense to care, a particularly important consideration for drugs requiring extensive and long-term monitoring. In the author's experience, patients may opt to go without monitoring because of cost, and may discontinue care or change to another provider because of the monitoring requirements. A further consideration regarding monitoring requirements is that clinical practices must remind patients, follow up on test results, and advise women if family-planning decisions are changed.

In summary, the evidence presented above demonstrates that GA fulfills many of the aspects important to patients: good tolerability, established long-term safety profile, pregnancy category, lack of associated serious adverse events, convenient dosing (particularly with the three-times weekly formulation), and no requirement for costly monitoring.

## 4. Perspectives and Conclusions

Over the past 20 years, there have been revolutionary changes in the treatment of MS. MRI–now the standard of care in the diagnosis of MS–allows earlier diagnosis, permitting earlier treatment. The numerous DMTs now available enable neurologists to individualize treatment and switch therapy if there are safety issues or lack of efficacy with first-line agents. The increased use of a multidisciplinary team approach and an improved awareness of the need to consider the patient's preferences and patient-reported outcomes (e.g., QOL) are further improvements in patient care, all of which facilitate a holistic approach for individuals living with MS.

The increase in the treatment armamentarium for MS provides more choice; however, treatment decisions are now more complicated than previously for physicians and patients. When coming to a decision to initiate long-term therapy for MS, the stakes are high for the patient owing to the recommendation to begin treatment early in the disease course, when the day-to-day clinical symptoms and disability may seem minor relative to the adverse effects of therapy. Indeed, the patient is required to take a “leap of faith” to begin therapy to prevent future disability and to continue therapy over the long term.

Patient perceptions regarding treatment efficacy and safety vary widely; therefore shared decision-making regarding DMT choice is the preferred approach. The reassuring long-term safety data, good tolerability, sustainability of effect over time, ability to self-administer, lack of requirement for safety monitoring, and extensive clinical experience with GA justify its position as a first-line treatment in early MS, and are critical factors underlying the continuing widespread use of GA more than two decades after its initial approval (Box 1). In addition, the 40 mg three-times weekly GA formulation provides benefits of less-frequent dosing and reductions in injection-site reactions. These factors, at least in part, explain why widespread use of GA has endured despite the availability of numerous alternative therapies.

A separate issue to consider is that access to MS-certified nurses through pharmaceutical company-funded patient-support programs provides a range of benefits for the patient. Such a program is available for GA. During treatment initiation, injection-technique training helps patients overcome any injection fear and manage injection reactions should they occur, and avoids patients stopping therapy prematurely. Once therapy is established, the patient–nurse rapport encourages adherence to therapy; nurses can also provide support during difficult periods. The Copaxone® patient-support program assists during the critical drug-initiation phase and through ongoing therapy and is likely to be a further factor supporting the continuing widespread use of GA.

The introduction of generic DMTs for MS is a significant concern for clinicians for numerous reasons. These include potential loss of the support programs and associated benefits, heritage and expertise of the product support teams, and manufacturing control, and the paucity of long-term data. This issue is not limited to GA. Introduction of other generic DMTs for MS in coming years will present new options but also new challenges for both patients and physicians, including concerns on areas such as manufacture, supporting data, and experience of use. Furthermore, generic competition will likely place additional insurance company restrictions on DMT choice for patients and physicians.

In conclusion, although Copaxone® has been marketed for more than 20 years, it is still a highly valued and widely used first-line treatment option for MS, despite the availability of numerous effective DMTs. The extensive long-term clinical experience, favorable safety profile and pregnancy rating, and convenient dosing regimen, together with persistent efficacy of Copaxone®, provide patients and physicians with confidence and peace of mind to begin treatment early and continue long term.

## Figures and Tables

**Box 1:**
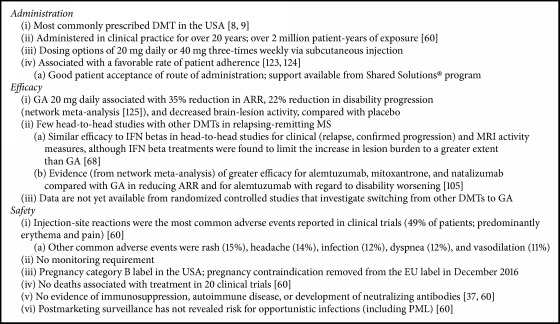
Summary of characteristics of branded glatiramer acetate in MS. ARR: annualized relapse rate; DMT: disease-modifying treatment; GA, branded glatiramer acetate; IFN: interferon; MS: multiple sclerosis; PML: progressive multifocal leukoencephalopathy.

**Table 1:** Long-term data and real-world data for GA in MS.

	**Study overview**						
**Study**	**Design ** **(Jadad score** ^a^ **)**	**Treatment**	**Patients**	**Duration, percent withdrawals**	**Key data**	**Comment**	**Reference**
**Pivotal studies**							

US Glatiramer Acetate Trial	Multicenter, randomized, PBO-controlled (5)	GA 20 mg SC QD vs PBO	Relapsing-remitting MS n=251	2 y, GA: 15.2 PBO: 13.5	29% reduction in ARR vs PBO: 0.59 vs 0.84 (*p*=0.007) Little difference between groups in progression to sustained disability (EDSS) Injection-site reaction most common AE	(i) US registration trial (ii) MRI endpoints not assessed	Johnson et al [39]

European/Canadian Glatiramer Acetate Study	Multicenter, randomized, PBO-controlled, double-blind (5)	GA 20 mg SC QD vs PBO	Relapsing-remitting MS n=239	9 mo, GA: 5.9 PBO: 5.8	29% reduction in total no. of T1-weighted enhancing lesions (mean reduction between groups: -10.8, *p*=0.003; GA: 25.96 vs PBO: 36.80) Consistent significant differences favoring GA across MRI endpoints: no. of new enhancing lesions, monthly change in enhancing lesion volume, change from baseline at 9 months in T2 lesion volume, no. of new lesions on T2-weighted imagesTreatment effects increased over time33% reduction in ARR for GA vs PBO: 0.81 vs 1.21 (*p*=0.012) No unanticipated AEs reported	(i) Study designed to assess effect of GA on MRI-measured disease activity and burden (monthly MRI scans); patients had to have ≥1 enhancing lesion (lesion levels relatively high at baseline)	Comi et al [40]

**Extension studies**							

US Glatiramer Acetate Trial	Prospective, open-label	GA 20 mg SC QD	Relapsing-remitting MS (n=232 received ≥1 dose [mITT]) (n=100 ongoing at 15 y; 74 ongoing at 20 y)	20 y, 15 y: 57 20 y: 68	*15-y timepoint* Ongoing patients: Mean GA exposure: 13.6 y; mean disease duration: 22 yARR reduced from 1.12 before starting GA therapy to 0.25 at 15-y timepoint57% had stable/improved EDSS scores (change ≤0.5 points) 65% had not transitioned to secondary progressive MS82% remained ambulatory without mobility aidsSafety consistent with previous shorter-term studies*20-y timepoint* Mean GA exposure: 19.3 y; mean disease duration: 27.3 yCumulative ARR: 0.224.3% relapse free through entire observation period53% had not transitioned to secondary progressive MS79.5% remained ambulatory without mobility aids (EDSS <6)	(i) Consistent low relapse rate and slow progression of disability (ii) Limitations of this study should be considered, e.g., prospective; patients with more aggressive disease may have dropped out (41% of withdrawals were owing to perceived disease progression or request to switch treatment); patients who remained in study may have had relatively mild MS (iii) Most common reason for drop-out from study was FDA approval of Betaseron® and desire to switch to an “FDA-approved” treatment (iv) No long-term safety issues identified (v) Only study of MS therapy with ≥10 y of continuous monitoring	Ford et al [41, 42]

European/Canadian Glatiramer Acetate Trial	Open-label extension	GA 20 mg SC QD (cross-over phase for PBO patients)	n=224	9 mo, GA: 4.0	Reduction in mean no. of Gd+ lesions was 46.5% for those receiving GA for full 18 mo vs 54% for those receiving GA after PBO35% fewer enhancements with continuous GA treatment (*p*=0.03) Corresponded to a mean of 6.6 fewer Gd+ lesions and 5.3 fewer new T2 lesions on quarterly scans for patients always receiving GA (e.g., during double-blind period and ongoing) vs those randomized to a delayed start (PBO during double-blind period)	(i) Reproducible reductions in mean no. of Gd+ lesions for patients originally receiving PBO and reductions maintained for those receiving GA for 18 months	Wolinsky et al [43]
						
	Open-label extension	GA 20 mg SC QD	Long-term follow-up group n=142 (73 received GA from study start)	5 y, GA: 34	Mean follow-up: 5.8 ySimilar MRI measures at 5 y between patients always receiving GA and those originally assigned to PBO (i.e., delayed start to GA) Fewer patients receiving GA required unilateral walking aids (6.9% vs 18.8% of those receiving PBO in double-blind phase of study) Percentage brain volume change at baseline and long-term follow-up significantly correlated with lesion load at entry	(i) Support for efficacy of early intervention (ii) Differences in trial design vs US extension study, i.e., patients in this trial had more aggressive MS (iii) High drop-out rate, which was related to the FDA/EMA approval of Betaseron® (as indicated above)	Rovaris et al [44]

**MRI study**							

Brain volume study	Retrospective	GA 20 mg SC QD, IFN*β*-1b 250 *μ*g SC EOD; IFN*β*-1a 30 *μ*g IM QW, untreated	Treatment-naïve, relapsing-remitting MS n=275	5 y	Significantly lower percentage change in brain volume loss from baseline to 5 y for GA -2.27% vs IFN*β*-1a -2.62% and IFN*β*-1b -3.21% (*p*<0.01) Reduced brain volume loss for all active treatments vs untreated (*p*<0.0001)	(i) GA-treated group experienced least loss of brain volume over 5 y (ii) Superior efficacy of GA vs IFN*β*-1a/b in slowing brain volume loss (iii) Study supports the importance of long-term observations for robust data on brain volume loss	Khan et al [45]

**Real-world data**							

—	Exploratory, retrospective US claims database analysis (PharMetrics Plus)	IFN/GA, fingolimod	Relapsing-remitting MS and history of relapse in past year n=525	540 days post-index continuous enrollment	ARR lower with fingolimod (0.32) vs either IFN or GA (both 0.64; *p*<0.0006)	(i) Limited data on patients' baseline characteristics and matching of cohorts limit conclusions	Bergvall et al [46]

SAME	Multicenter, non-interventional, retrospective cohort study	IFN*β*-1a 30 *μ*g IM, IFN*β*-1a 22/44 *μ*g SC, IFN*β*-1b 250 *μ*g SC, GA 20 mg SC at standard doses	Relapsing-remitting MS or CIS n=546	2 y	Comparable ARR at 1 y and 2 yYear 1 (% patients with ≥1 relapse): GA: 18%, IFN*β*: 16–24%Year 2: GA: 22% vs IFN*β*: 23–27%EDSS changes between Year 1 and Year 2 were comparable between groups	(i) Patient cohorts were not matching at baseline (ii) Not stated in study report whether the study was powered to demonstrate EDSS change	Gobbi et al [47]

XPERIENCIA-5	Multicenter, retrospective	GA for ≥5 y	Relapsing-remitting MS n=149	Up to 9 y	ARR ranged from 1.5 pretreatment to 0.2–0.3 over 9 y of treatment89.9% patients free from disability progression at Year 5 and 85.7% remained so at Year 975.2% of patients showed long-term absence of disability progression for ≥5 consecutive years92.6% remained ambulatory without mobility aidsReductions in Gd+ T1-weighted lesions (*p*<0.5) and no. of new T2 lesions (*p*=NS) from baseline to last follow-up	(i) Consistent with decline in disability progression in pivotal study extensions (detailed above) (ii) Value of data from later years reduced due to decreasing patient numbers in Years 7–9 (Year 7 n=61, Year 9 n=21) (iii) Supports long-term sustained efficacy for GA in stabilizing disease (low ARR and disability [EDSS] progression) (iv) Safety data not reported (v) Supports long-term patient acceptance of injectable therapy	Arnal-García et al [48]

MSBase Study	Propensity score-matched analysis of MSBase registry	IFN*β*-1a IM, IFN*β*-1a SC, IFN*β*-1b, GA	n=3326	Median follow-up: 3.7 y (IQR 2.2–6.3 y)	Lower relapse rate with GA treatment vs IFN*β*-1a IM or IFN*β*-1b (observed mean differences 0.15–0.16; *p*<0.001) Higher proportion of relapse-free patients with GA vs IFN*β*-1a IM or IFN*β*-1b (hazard ratio 1.36 and 1.48, respectively; *p*≤0.02) No differences in 1-y confirmed progression of disability over 10 y	(i) Higher proportion of patients were relapse-free with GA treatment compared with IFN*β*-1a/b (ii) Safety data not reported	Kalincik et al [49]

AE: adverse event; ARR: annualized relapse rate; CIS: clinically isolated syndrome; EDSS: Expanded Disability Status Scale; EMA: European Medicines Agency; EOD: every other day; FDA: Food and Drug Administration; GA: branded glatiramer acetate; Gd+: gadolinium-enhancing; IFN: interferon; IM: intramuscular; IQR: interquartile range; mITT: modified intent to treat; MRI: magnetic resonance imaging; MS: multiple sclerosis; NS: not significant; PBO: placebo; QD: once daily; QW: once weekly; SC: subcutaneous.

^a^Jadad scores for randomized controlled studies [50].

**Table 2:** Active-comparator clinical studies with GA in MS.

	**Study overview**						
**Study**	**Design (risk of bias)** ^a^	**Treatment**	**Patients**	**Duration, Percent withdrawals **	**Key data**	**Comment**	**Reference**
REGARD	Multicenter, randomized, comparative, parallel-group, open-label, assessor-blinded (low)	GA 20 mg SC QD vs IFN*β*-1a 44 *μ*g SC TIW	Relapsing-remitting MS n=764	96 wk, IFN: 21 GA: 14	No significant difference between groups for time to first relapse (HR 0.94 [95% CI 0.74 to 1.21]; *p*=0.64) No significant differences between groups for no. and change in volume of T2-active lesions or for change in volume of Gd+ lesionsFewer Gd+ lesions in patients treated with IFN*β*-1a (0.24 lesions/patient/scan vs 0.41 for GA; *p*=0.0002) Significantly greater reduction in brain volume with IFN*β*-1a (-1.24%) vs GA (-1.07%; *p*=0.018)	(i) Relapse rates in the trial were lower than predicted, thus limiting prediction of clinical superiority. This may be a fault of the trial design due to a limited number of patient contacts and also site selection (ii) Brain volume data indicate that brain atrophy precedes/is correlated with disability progression	Mikol et al. [51]

BECOME	Randomized, single-blind (low)^b^	GA 20 mg SC QD vs IFN*β*-1b 250 *μ*g EOD SC	Treatment-naïve relapsing-remitting MS or CIS n=75	2 y, IFN: 19.4 GA: 10.3	Similar median [75th percentile] combined active lesions/patient/scan (GA: 0.58 [2.45], IFN*β*-1a: 0.63 [2.76]) No differences in new lesions or clinical relapses over 2 y	(i) Similar MRI and clinical activity following GA or IFN*β*-1a treatment (ii) Greater subclinical disease activity than previously observed was also reported in this study. However, these data should be interpreted with caution as the small trial size limits conclusions	Cadavid et al [52]

BEYOND	Multicenter, randomized, parallel-group (low)	GA 20 mg SC QD vs IFN*β*-1b SC (250 *μ*g or 500 *μ*g) EOD	Treatment-naïve relapsing-remitting MS n=2244	2–3.5 y IFN 250 *μ*g: 11.6 IFN 500 *μ*g: 17.9 GA: 15.8	No differences between groups in relapse risk, disability (EDSS) progression, T1-hypointense lesion volume, or normalized brain volumeSignificant decrease in T2 lesion volume observed with both IFN*β*-1b groups vs GA at Year 1 but not during Years 2 and 3	(i) Similar clinical efficacy for GA and IFN*β*-1b (ii) GA and IFN*β*-1b have different adverse-event profiles; however, overall tolerability is similar	O'Connor et al [53]

CONFIRM	Multicenter, randomized, double-blind^c^ (low)	GA 20 mg SC QD vs DMF 240 mg BID PO, 240 mg TID PO,^d^ or PBO PO	Relapsing-remitting MS n=1417	2 y, DMF BID: 29.5 DMF TID: 27.8 GA: 24.6 PBO: 35.5	ARR significantly lower vs PBO with all active treatments (GA: 0.29; DMF BID: 0.22, TID: 0.20; PBO: 0.40) No significant differences in reduction of disability progression for GA or DMF vs PBOPost hoc comparison revealed a significantly greater treatment effect for DMF vs GA for ARR (TID dose), no. of new or enlarging hyperintense lesions on T2-weighted images (both BID and TID), and T1-weighted images (TID dose)	(i) Apparent convenience of oral therapies complicated by issues including adverse events and monitoring requirements (ii) Study was not powered to test the superiority or noninferiority of DMF vs GA. Post hoc comparisons are therefore not informative (iii) Markedly higher drop-out rate with oral DMF vs GA owing to adverse events	Fox et al [54]

CombiRx	Double-blind, randomized, placebo-controlled (low)	IFN*β*-1a 30 *μ*g IM weekly GA 20 mg SC QD Combination IFN*β*-1a + GA	Relapsing-remitting MS n=1008	3 y, IFN: 22.4 GA: 13.9 Combination: 20.4	Combination was not superior to GA for ARR or 6-mo confirmed progression of disability (EDSS) Compared with IFN, both combination (25%; *p*=0.022) and GA alone (31% reduction, *p*=0.027) significantly improved ARR; no differences in disability (EDSS) progressionCombination superior to IFN or GA alone in reducing new lesion activity and accumulation of total lesion volumesNo unexpected safety issues observed with combination	(i) Similar results were observed after a further 4 y of follow-up (ii ) The reduction in MRI activity in the 3-y study did not result in a later clinical advantage at 7 y	Lubin et al. [55, 56]

ARR: annualized relapse rate; BID: twice daily; CI: confidence interval; CIS: clinically isolated syndrome; DMF: dimethyl fumarate; EDSS: Expanded Disability Status Scale; EOD: every other day; GA: branded glatiramer acetate; Gd+: gadolinium-enhancing; HR: hazard ratio; IFN: interferon; IM: intramuscular; MRI: magnetic resonance imaging; MS: multiple sclerosis; PBO: placebo; PO: oral; QD: once daily; SC: subcutaneous; TID: three-times daily; TIW: three times per week.

^a^Based on assessment of risk of bias with regard to randomization; baseline characteristics; blinding; withdrawal/discontinuation; outcome selection, reporting, and other sources of bias; and statistical analysis [57]. ^b^Low risk of bias for all aspects other than randomization (not reported) [57]. ^c^Patients receiving GA were aware of their treatment allocation. ^d^Dose not Food and Drug Administration approved.
